# Brain Functional Network Alterations in Adolescents With Major Depressive Disorder and Suicidal Attempt: A Resting-State fMRI Study

**DOI:** 10.31083/AP48336

**Published:** 2026-04-22

**Authors:** Wei Peng, Yujie Yang, Zhenhong Liao, Gaoyuan Liu, Ping Liu, Feifei Zhang

**Affiliations:** ^1^Department of Radiology, Deyang People's Hospital, 618000 Deyang, Sichuan, China; ^2^Department of Psychosomatics, Deyang People's Hospital, 618000 Deyang, Sichuan, China; ^3^Department of Radiology, First Hospital of Shanxi Medical University, 030001 Taiyuan, Shanxi, China

**Keywords:** adolescent, brain, depression, magnetic resonance imaging, suicide

## Abstract

**Background:**

The neurobiological mechanism underlying suicidal attempts in depressive adolescents remains unknown. This study investigated brain functional network changes in adolescent depression with suicidal attempt.

**Methods:**

First episode, treatment naïve adolescent patients with depression and suicidal attempts, and healthy adolescents matched for age and sex were enrolled. Resting-state functional magnetic resonance images were obtained and whole-brain functional matrices were constructed to analyze functional connectivity differences between the two groups. The graph theory method was used to analyze topological alterations in brain regions with altered functional connectivity. Correlations were analyzed between functional parameters and clinical characteristics.

**Results:**

A total of 47 patients and 33 healthy adolescents were included. Depressed adolescents with suicidal attempts showed lower functional connectivities than did healthy controls in critical brain regions, mainly located in the sensorimotor network, default mode network, cognitive control network, visual network and cerebellum. Graph theory analysis revealed a decreased degree centrality in the right precentral gyrus of patients. Correlation analyses identified a negative association between depressive symptom severity and functional connectivity in the left supplementary motor cortex-inferior parietal lobule in the patient group.

**Conclusions:**

This study revealed functional abnormalities in depressed adolescents with suicidal attempts in critical brain networks that were responsible for emotional regulation, cognitive process and behavioral controls, and suggested critical neurobiological evidence of suicidality in depression.

## Main Points

1. Impaired functional connectivity within and between major brain networks (sensorimotor network [SMN], default mode network [DMN], cognitive control network [CCN], visual network [VN]) may underlie the integration of emotional, sensory, and cognitive disruptions in adolescent depression that contribute to suicidality.

2. Decreased information transfer efficiency was observed in the patients’ right precentral gyrus, with a reduction in its degree centrality.

3. The right precentral gyrus may be a key region in suicidality, characterized by abnormalities in both functional connectivity and topography.

4. Reduced functional connectivity in the left supplementary motor cortex-inferior parietal lobule might be indicative of depressive symptom severity.

## 1. Introduction

Adolescence is a critical period for psychosomatic development, and mood disorders, such as major depressive disorder (MDD), are likely to occur [1]. Compared to adult MDD, adolescent MDD has a higher risk of suicide, and suicide is the second most common cause of mortality in adolescents [2], causing great damage to family and society. The rate of suicide among depressive adolescents was reported to possibly reach up to 60% among inpatients [3]. However, the neuropathophysiology of suicide in depression still remains unclear.

Multi-modal magnetic resonance imaging (MRI) has been widely applied to investigate brain structure and function non-invasively, and has revealed critical neurobiological mechanisms for psychiatric disorders, such as depression, anxiety and schizophrenia [4, 5]. Previous functional MRI (fMRI) studies have reported decreased functional activity in the frontal cortex and precuneus in depressed adolescents with suicidal behaviors, which was associated with cognitive impairment and mood dysregulation, suggesting potential pathophysiology for suicide in adolescent MDD [6, 7]. Despite abnormal regional activity, functional studies also found disrupted connectivities in critical brain regions of MDD patients with suicidality, such as the prefrontal cortex, the cingulate cortex and the precuneus, which are responsible for cognition and emotional regulation [8].

Besides functional activity and connectivity, a recent study has also demonstrated that brain neurons presented topological characteristics that integrate and segregate information in an effective approach [9]. In this respect, the brain is a network that consists of nodes and edges. The nodes represent cortical and subcortical regions, while the edges represent connections between these regions that are responsible for information transformation [10]. This conceptualization can aid in the exploration of neural functional mechanisms of psychiatric disorders and behavioral problems, and has revealed altered topography in critical brain regions and networks underlying psychiatric diseases. Examples are greater randomization in functional brain networks of schizophrenia and greater regularization in MDD, which could help to investigate neural mechanisms of psychiatric conditions [11].

Based on previous studies, we hypothesized that suicidality in adolescent MDD might be related to functional aberrations in critical brain networks responsible for cognition and emotion, such as the default mode network (DMN) and the cognitive control network (CCN). Therefore, in this study, we focused on depressed adolescents with suicidal attempts, identified brain functional connectivity changes and topographic alterations, to comprehensively explore possible neural mechanisms and potential imaging biomarkers of suicidality in adolescent depression.

## 2. Materials and Methods

Patients who were diagnosed with MDD, had suicidal attempts, and met all study criteria were recruited consecutively from both inpatient and outpatient settings at Deyang People’s Hospital, and were consecutively enrolled between August 2021 and April 2025. Experienced radiologists reviewed the conventional brain magnetic resonance (MR) images to rule out structural abnormalities. The diagnosis of MDD was made based on the Structured Clinical Interview for the Diagnostic and Statistical Manual of Mental Disorders-V (DSM-5) (SCID-5), and every patient had shown at least one suicidal attempt during the illness duration. Symptom severity was evaluated using the 17-item Hamilton Depression Rating Scale (HAMD). The healthy control group was recruited from the community through advertisements; they were then demographically matched to the patients. All healthy controls were screened by psychiatrists to confirm the absence of any personal history of neuropsychiatric illness, brain injury, suicidal attempt or ideation, as well as no family history of suicide or major mental illness (e.g., depression) in first-degree relatives. The exclusion criteria were a history of psychosis, neurodevelopmental diseases such as autism or attention deficit hyperactivity disorder, comorbid mental disorders such as trauma- or stressor-related disorders, significant neurological or medical illness, electroconvulsive or psychiatric drug therapy, alcohol or substance abuse or dependence, MRI contraindications, and age <10 or >19 years.

### 2.1 MRI Scanning

All participants were scanned using a 3.0 T MR scanner (Philips Ingenia, Amsterdam, Netherlands) with a 15-channel head coil. Participants were fitted with soft earplugs, comfortably positioned in the coil, and instructed to remain still. Head motion was further minimized using foam pads for immobilization. Resting-state fMRI images were obtained with an echo planar imaging (EPI) sequence with the following parameters: Repetition Time/Echo Time (TR/TE) = 2000/30 ms, flip angle (FA) = 90°, field of view (FOV) = 224 × 224 mm^2^, 33 axial slices with 3.5 mm slice thickness, matrix = 64 × 63, 240 volumes. High-resolution 3D T1-weighted images were acquired using a fast field echo (FFE) sequence with the parameters as follows: TR/TE = 7.3/3.3 ms, FA = 8°, 360 sagittal slices with an acquired voxel size of 1 × 1 × 1 mm^3^, FOV = 240 × 236 mm^2^ and data matrix = 240 × 237, yielding a reconstructed voxel size of 0.5 × 0.5 × 0.5 mm^3^.

### 2.2 Data Preprocessing

To ensure data quality, experienced neuroradiologists reviewed all whole-brain images to exclude both those with visible structural abnormalities and those compromised by head motion or other artifacts. MRI data were then preprocessed using GRETNA (v2.0.0) software (Beijing, China, http://www.nitrc.org/projects/gretna) [12]. To allow for magnetization stabilization, the first 10 volumes were discarded. The remaining images were then processed for slice timing correction and motion realignment. Participants with head motion exceeding 3 mm in translation or 3° in rotation were excluded from subsequent analysis. Subsequently, images were normalized to the Montreal Neurological Institute (MNI) space by using the diffeomorphic anatomical registration through exponentiated Lie algebra (DARTEL) method. Then, the normalized images were temporally detrended, temporal band filtered with 0.01~0.08 Hz, and smoothed with a 4-mm full-width at half-maximum Gaussian kernel. Finally, to remove potential confounding signals, the Friston-24 parameters, global white matter signal, and cerebrospinal fluid signal were included as regressors. In addition, framewise displacement parameters were used to ensure that groups were comparable.

### 2.3 Brain Network Analysis

The brain was divided into 264 regions using the Power 264 template, which is a common brain functional atlas [13]. Each region is considered a network node. The Pearson correlation coefficient between each node pair was calculated. The Fisher-Z transformation was used to normalize the Pearson correlation coefficient as the edge, yielding a 264 × 264 correlation matrix for functional connectivity (FC) of each subject. The independent two sample *t* test was used for FC comparisons, with the threshold of uncorrected *p* < 0.001 and false discovery rate (FDR) <0.05 for multiple comparisons correction.

Graph theory method was used to analyze topographic characteristics in regional brain areas with altered FC, including clustering coefficient (Cp), local efficiency (Eloc), shortest path length (Lp), degree centrality (Dc), and betweenness centrality (Bc). To ensure meaningful network connectivity and to facilitate the estimation of small-world organization, the sparsity threshold used in our study was set at 0.05–0.4, with an interval of 0.01, based on calculations [14] and a previous study [15] to ensure network organization integrity. The area under the curve (AUC) for each nodal parameter was calculated across each threshold for further independent two sample *t* test comparisons. The FDR correction procedure was used for correction of the multiple comparisons, with the threshold of corrected *p* < 0.05.

### 2.4 Statistical Analysis

The statistical comparisons were conducted with SPSS 19.0 software (SPSS, Inc., Chicago, IL, USA). Age and sex were compared with a two-sample independent *t* test and chi-square test, respectively, since the age of the participants presented a normal distribution, whereas the sex data were categorical. Altered FCs and topographic parameters were extracted for further correlation analysis. Pearson correlation analysis was conducted to evaluate associations between depressive severity and brain functional alterations in patients, since the data distributions were normal. In addition, nonparametric Spearman correlation analysis was performed to assess the relationships between illness duration and brain functional changes, due to the distribution of illness duration being non-normal. Subgroup analyses were conducted for FCs and topographic parameters in males and females, respectively. Owing to the limited sample size for males, data are presented as individual distributions; in contrast, the two-sample independent *t* test was employed for the analysis of female participants.

## 3. Results

In total, 64 adolescents with first-episode, treatment-naïve MDD and a history of suicidal attempts, along with 48 healthy controls, met the study criteria and were enrolled. Among them, 1 healthy subject and 4 patients were excluded due to the poor image quality after the visual check by experienced radiologists. Subsequently, 14 healthy subjects and 13 patients were excluded due to maximal translation of more than 3 mm or maximal rotation of more than 3° after motion realignment. Hence, 33 healthy subjects and 47 patients were finally included for final analysis. The demographic and characteristics are shown in Table 1. No significant differences were found between patients and healthy controls regarding age, sex, or handedness. The patient group had a mean HAMD score of 22.8. There was no significant difference in framewise displacement between the two groups, ensuring the groups were comparable for the following analyses.

**Table 1.** **Demographic and characteristics of patients and healthy controls**.

Characteristics	Suicidal MDD (n = 47)	HC (n = 33)	*p* value
Age (mean years ± SD)	15.8 ± 2.8	14.9 ± 2.2	0.13
Gender (male/female)	9/38	9/24	0.39
Handedness (right/left)	47/0	33/0	>0.99
Illness durations (months)	4.4 ± 3.6		
HAMD score	22.8 ± 3.1		
FD (mean ± SD)	0.140 ± 0.059	0.139 ± 0.084	0.94

Abbreviations: FD, framewise displacement; HAMD, Hamilton Depression Rating Scale; HC, healthy control; MDD, major depressive disorder; SD, standard deviation.

### 3.1 Functional Connectivity Results

**Fig. 1.**
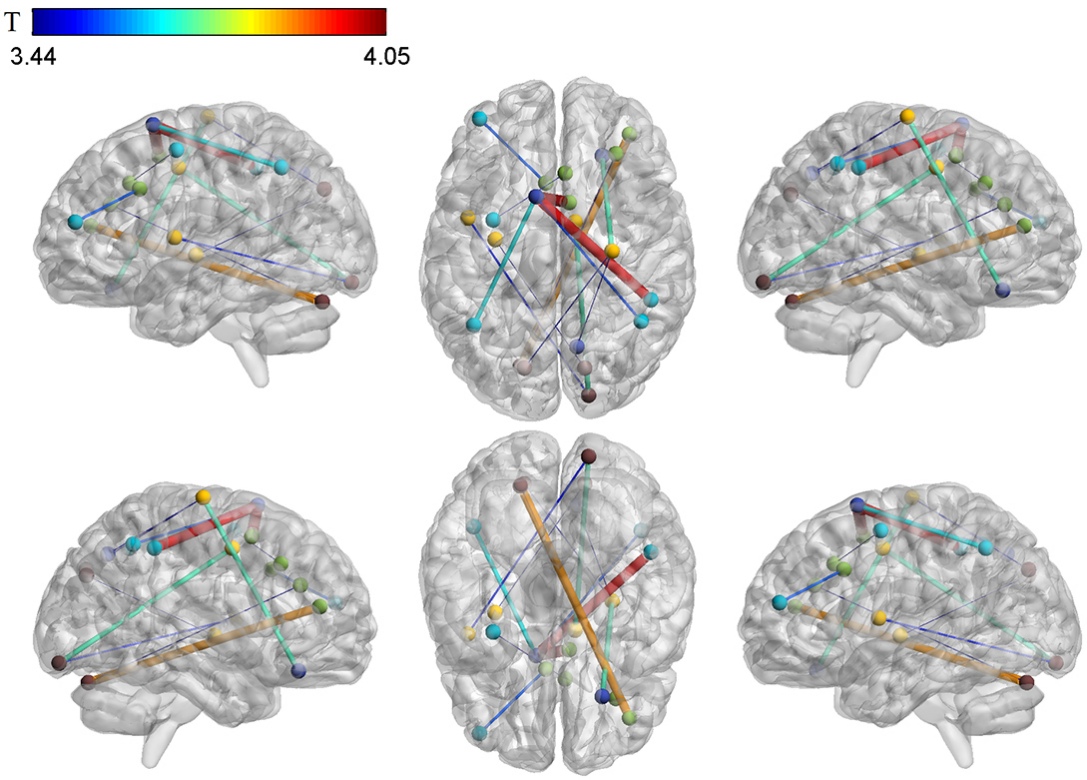
**Differences in brain functional connectivity between depressed adolescents with suicidal attempts and healthy subjects**. The color bar shows the range of T values.

**Fig. 2.**
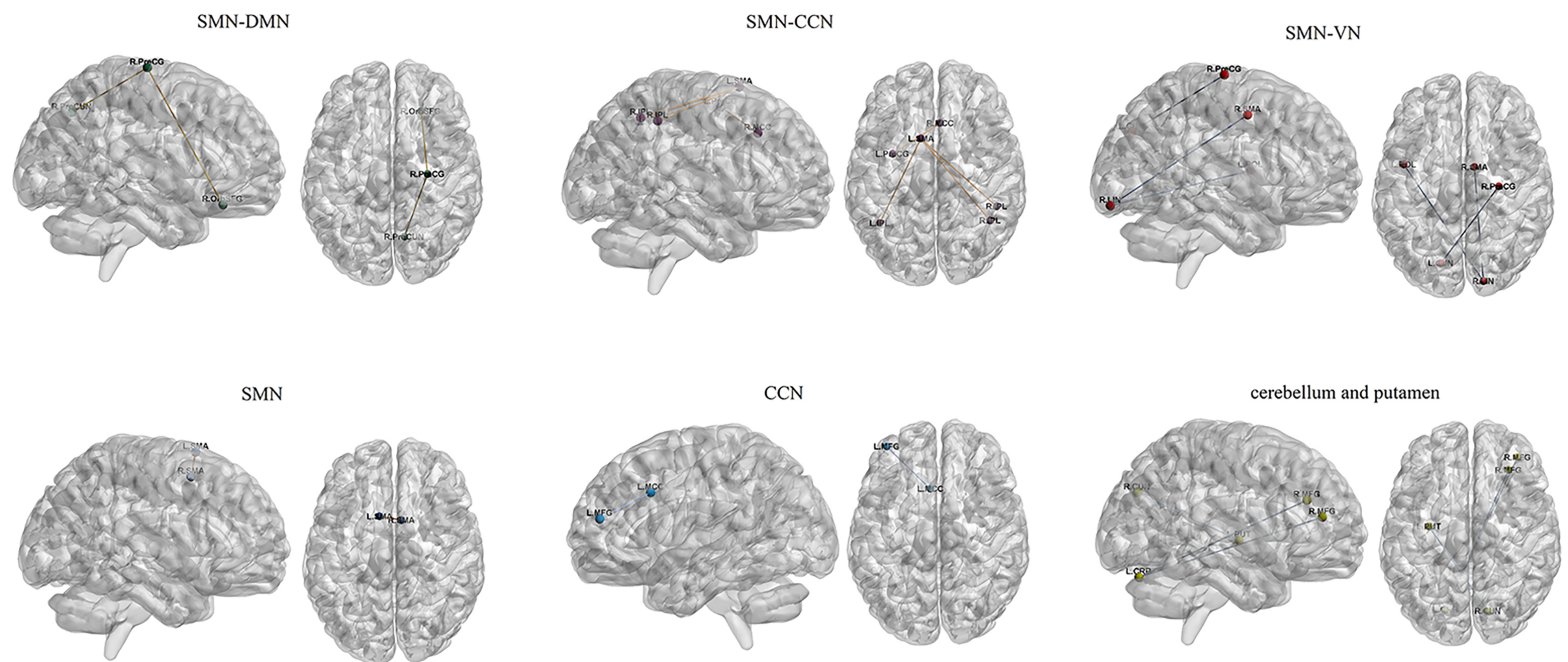
**Functional connectivity alterations in brain networks of adolescent depression with suicidal attempts**. Abbreviations: CCN, cognitive control network; CRB, cerebellum; CUN, cuneus; DMN, default mode network; IPL, inferior parietal gyrus; LIN, lingual gyrus; MCC, middle cingulate cortex; MFG, middle frontal gyrus; OrbSFG, orbital superior frontal gyrus; PreCG, precentral gyrus; PreCUN, precuneus; PUT, putamen; ROL, rolandic operculum; SMA, supplementary motor area; SMN, sensorimotor network; VN, visual network.

**Fig. 3.**
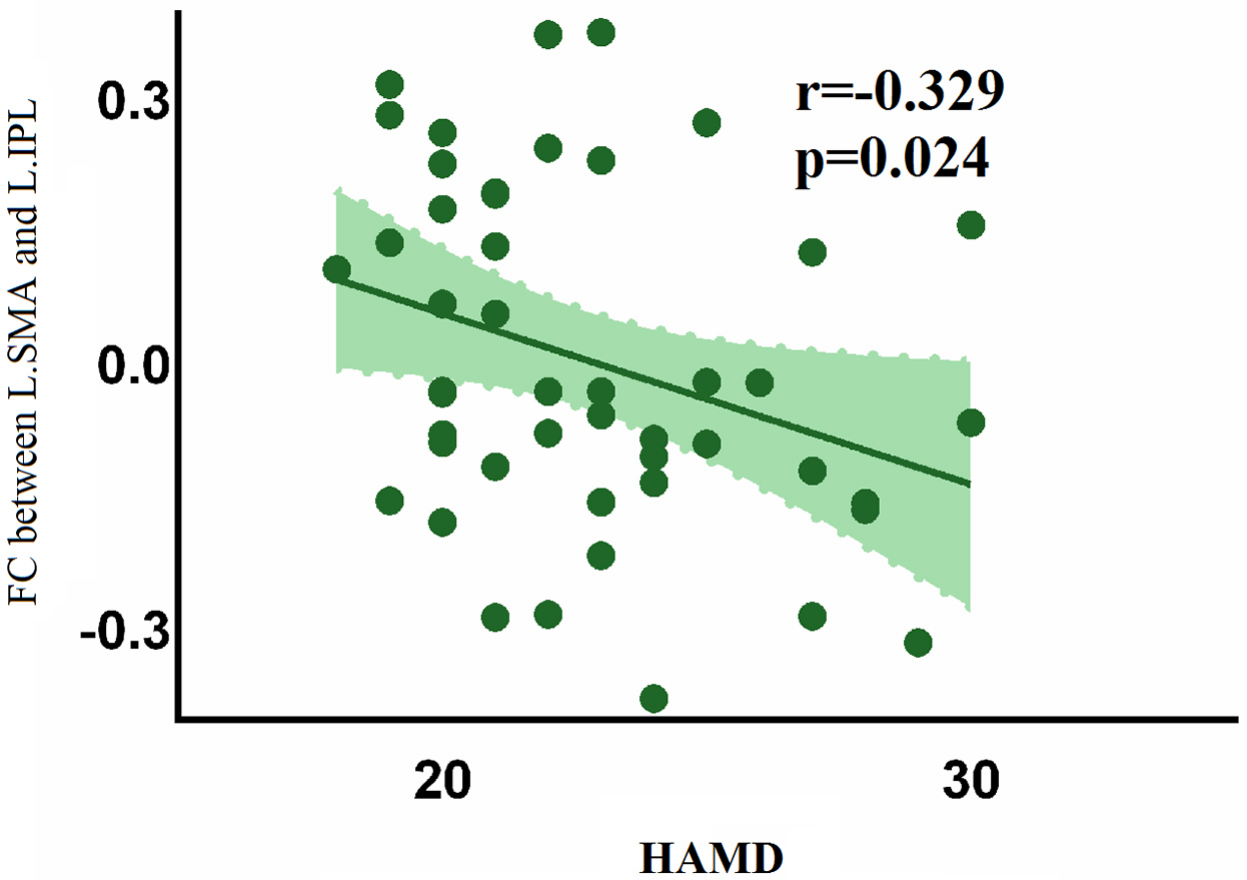
**Negative association between HAMD and FC in the left supplementary area-left inferior parietal lobule in patient group**.

Depressed adolescents with suicidal attempts presented lower FCs than did healthy subjects, between several critical brain regions, including the right precentral gyrus-right superior orbital frontal gyrus, the right precentral gyrus-bilateral precuneus, the right supplementary motor cortex-right lingual gyrus, the right supplementary motor cortex-left supplementary motor cortex, the left rolandic operculum-right lingual gyrus, the left middle frontal gyrus-left middle cingulum cortex, the left supplementary motor cortex-bilateral inferior parietal lobule, the right cuneus-left putamen, the right middle frontal gyrus-left cerebellum, the left precentral gyrus-right middle cingulum cortex (Table 2, Fig. 1). These brain regions are mainly located in the sensorimotor network (SMN), DMN, CCN, visual network (VN) and cerebellum (Fig. 2). Correlation analysis further revealed that the FC between the left supplementary motor cortex and the left inferior parietal lobule was negatively associated with the HAMD score in patient group (Fig. 3), but it was modest and did not survive after FDR correction. In the subgroup analyses, results for female participants were completely consistent with the results of the whole-group analysis (see **Supplementary Table 1**). Results for the male subgroup were largely consistent, except that reduced FC was not observed in several regions among patients (see **Supplementary Fig. 1**).

**Table 2.** **Differences of functional connectivity between patients and healthy controls**.

Anatomical regions		FC (Mean ± SD)		T value
		Suicidal MDD	HC	
Precentral_R	Frontal_Sup_Orb_R	0.06 ± 0.18	0.23 ± 0.19	3.70
Precentral_R	Precuneus_R	0.12 ± 0.23	0.32 ± 0.27	3.46
Supp_Motor_Area_R	Lingual_R	0.07 ± 0.21	0.22 ± 0.15	3.70
Precentral_R	Cuneus_L	0.12 ± 0.19	0.29 ± 0.25	3.47
Supp_Motor_Area_R	Supp_Motor_Area_L	0.01 ± 0.20	0.23 ± 0.23	4.05
Rolandic_Oper_L	Lingual_R	0.03 ± 0.17	0.16 ± 0.14	3.52
Cingulum_Mid_L	Frontal_Mid_L	0.30 ± 0.27	0.49 ± 0.21	3.56
Supp_Motor_Area_L	Parietal_Inf_R	–0.09 ± 0.20	0.08 ± 0.18	3.99
Supp_Motor_Area_L	Parietal_Inf_R	–0.04 ± 0.22	0.13 ± 0.17	3.55
Supp_Motor_Area_L	Parietal_Inf_L	0.01 ± 0.19	0.17 ± 0.17	3.66
Cuneus_R	Putamen_L	0.15 ± 0.19	0.30 ± 0.18	3.44
Frontal_Mid_R	Cerebellum_L	0.16 ± 0.13	0.29 ± 0.19	3.46
Cingulum_Mid_R	Precentral_L	0.20 ± 0.26	0.38 ± 0.21	3.45
Frontal_Mid_R	Cerebellum_L	0.09 ± 0.16	0.23 ± 0.14	3.89

Abbreviations: Cingulum_Mid, middle cingulate cortex; FC, functional connectivity; Frontal_Mid, middle frontal gyrus; Frontal_Sup_Orb, orbital superior frontal gyrus; L, left; Lingual, lingual gyrus; Parietal_Inf, inferior parietal lobule; Precentral, precentral gyrus; R, right; Rolandic_Oper, rolandic operculum; Supp_Motor_Area, supplementary motor area.

### 3.2 Graph Theory Analysis Results

**Fig. 4.**
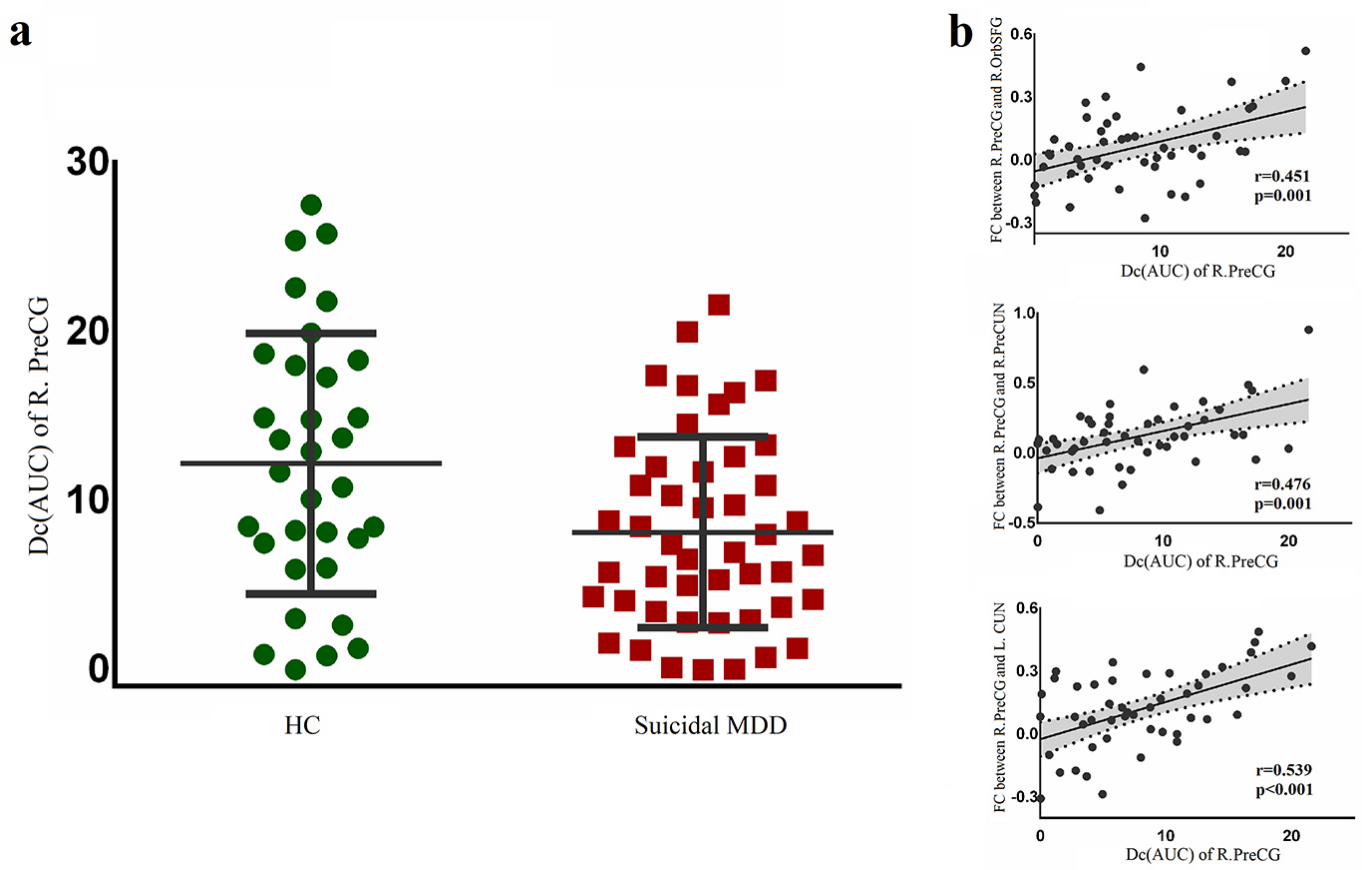
**Dc changes in the right precentral gyrus of patients**. (a) presented a reduced AUC of Dc in the patient group compared with the healthy group. (b) showed positive correlations between AUC of Dc in the right precentral gyrus and FCs in the right precentral gyrus-right superior orbital frontal gyrus, right precentral gyrus-right precuneus, and right precentral gyrus-left cuneus. Abbreviations: AUC, area under the curve; Dc, degree centrality.

Graph theory analysis revealed that the AUC values of Dc in the right precentral gyrus were significantly lower in patients than in healthy subjects (*p* = 0.008, Fig. 4a), whereas other nodal parameters did not differ significantly between the two groups (*p* > 0.05). In addition, Dc in the right precentral gyrus was positively correlated with FC in the right precentral gyrus-right superior orbital frontal gyrus, the right precentral gyrus-right precuneus, the right precentral gyrus-left cuneus after FDR correction, but was not significantly correlated with HAMD score or illness duration in patient group (Fig. 4b). The subgroup analyses for both females and males were consistent with the overall group result, showing decreased Dc in the right precentral gyrus in patients (see **Supplementary Fig. 2**).

## 4. Discussion

Our study indicated that depressed adolescents with suicidal attempts mainly showed impaired FCs among SMN, DMN, CCN and cerebellum. In addition, decreased information transfer efficiency was identified in the right precentral gyrus in patients, which was mainly related to disrupted FC with DMN. These findings possibly suggested a neural functional basis for suicidal attempt in adolescent depression.

The precentral gyrus, supplementary motor cortex and rolandic operculum are critical regions of the SMN that are responsible for sensory and movement control. Therefore, functional abnormalities in these regions might be related to impulsive behaviors like suicidal attempts [16, 17, 18]. Our findings were consistent with a previous study that suggested that, functional and structural aberrations in the precentral gyrus were associated with suicidal attempts in depression [19]. In addition, in the previous study, changes in functional activity in the precentral gyrus were correlated with suicidal ideation severity in depressive patients [19], implying a critical role of altered precentral gyrus activity in suicidality. Tymofiyeva *et al*. [20] reported that, compared to depressed patients without suicidal attempt, depressed patients with suicidal attempts had lower FC between the supplementary motor cortex and putamen, suggesting that altered function in the supplementary motor cortex might be one of the potential neurobiological mechanisms of suicide in depression. Altered gray matter volumes in the rolandic operculum were also reported in depression; this could also support our finding of abnormal FC in this brain region, suggesting another important possible mechanism for suicide in MDD [21]. Therefore, functional dysregulation in the SMN indicated the neuropathophysiological potential for suicidal attempt in adolescent depression.

The orbital frontal gyrus and precuneus are critical components of the DMN, which is responsible for emotional regulation and self-reference [22, 23]. Damages in these areas might influence self-regulation and cognitive ability, which could increase the risk of suicidality [24]. Recent functional studies reported that abnormal activity in the orbital frontal gyrus could help to distinguish MDD with suicidal ideation from non-ideators [25], and the activation in orbital frontal cortex was negatively correlated with suicidal ideation severity after treatment in MDD [26], suggesting altered function in orbital frontal cortex might contribute to suicidality in MDD. In addition, altered function in the precuneus was reported to be associated with suicidal risk in drug naïve MDD [27]. Moreover, abnormal FCs and activity in the DMN have also been reported to underlie neural mechanisms of suicidal activity in MDD [22, 28]. Besides, an electroencephalography study also confirmed that DMN dysfunction was associated with active suicidal ideation in adolescents [29]. Hence, consistent with previous findings, our results implied the critical role of DMN dysfunction in suicidal attempts.

Our study identified disrupted FCs in the CCN of patients, which is mainly located in the middle frontal gyrus, the middle cingulate cortex and the inferior parietal lobule, suggesting that cognitive impairment might contribute to suicidality in depression [18]. A brain structural study reported reduced cortical folding in the middle frontal gyrus of depressed adolescents with suicidal attempts [30], thereby providing structural evidence for our functional result. Also, Cheng *et al*. [31] showed that abnormal function in the middle frontal gyrus was associated with impulsive decision-making in depressed adolescents, thus increasing the risk of suicidal behaviors. Furthermore, a neuroimaging meta-analysis of suicide indicated that functional activity in the middle cingulate cortex was damaged in suicidal individuals [21]. One large-sample mega-analysis on adult MDD indicated that patients with suicidal attempts showed significant structural changes in the inferior parietal cortex compared with patients without suicide and with healthy subjects [32], which was consistent with our findings and suggested that the inferior parietal cortex abnormalities might be the imaging biomarker for suicide in depression.

The cuneus and fusiform gyrus are critical for visual cognition [33], and we found that abnormal FCs of the VN might be related to suicidal attempts in adolescent MDD. A previous diffusion tensor imaging study identified alterations in white matter fibers in the occipital cortex of suicidal attempters, which also supported our results [34]. Moreover, another meta-analysis demonstrated that decreased functional activity in the fusiform gyrus was associated with suicidal behaviors in MDD [35], and abnormal functional activity in the cuneus was also identified in high-suicidality depressed patients versus low-suicidality depression and healthy subjects [36], emphasizing the essential role of altered VN function in the neurobiological mechanisms of suicide.

We also identified altered FCs in the cerebellum and putamen in adolescent MDD with suicidal attempts. Traditionally, the cerebellum and putamen are responsible for movement regulation, while neuroimaging studies have demonstrated that the cerebellum and putamen also participate in cognitive processes and emotional regulation, such as decision-making and goal-directed behaviors [37, 38]. Studies on suicidality revealed that abnormal gray matter volume and functional activity in the cerebellum were related to suicidal ideation and actions [39, 40], and increased metabolism in the cerebellum was related to decreased suicidal ideation in adults [36], suggesting neuroimaging evidence for abnormal cerebellum activity in suicidal mechanisms. Gifuni *et al*. [37] found gray matter abnormalities in the putamen of adolescent MDD with suicide, compared to non-suicidal patients and healthy subjects. Additionally, a previous functional study also found altered FCs in the putamen-supplementary motor cortex and the putamen-superior frontal gyrus in depressed adolescents with suicidal attempts [20]. These findings were consistent with our results and suggested that dysfunction of the cerebellum and putamen could be a possible neurobiological mechanism for suicide.

Recent studies demonstrated that altered FCs between DMN and CCN were associated with impulsivity and hopelessness severity in depressed adolescents with suicidal attempts [23], and altered FCs of these networks could help distinguish suicidal attempters from non-suicidal patients with mood disorders and from healthy subjects [41]. Besides, a large-scale, transdiagnostic task-fMRI study (including mood, anxiety, and stress disorders) found that suicidal participants exhibit decreased intra- and inter-network connectivity among the SMN, DMN, and VN, suggesting potential neural signatures of general psychopathology pertinent to suicidality [42]. Furthermore, transcranial magnetic stimulation therapy and cognitive behavioral therapy for suicidal MDD were reported to be effective in reducing suicidal ideation and in clinical symptom remission, coinciding with a restoration of abnormal functional connectivity and activity among DMN, CCN, SMN and VN [43, 44], indicating possible neural target for innovative treatment instead of common antidepressants, which was reported to increase the risk of suicide among patients and may not be suitable for depressed adolescents with suicide [45]. Taken together, these findings were consistent with our results, and demonstrated that abnormal functional conditions in the DMN, SMN, CCN and VN could be crucial neural mechanisms for suicidal attempt in adolescents, not only for general depression effects. Besides, we enrolled first episode and treatment naïve patients in order to exclude the impact of medication and recurrence. We felt that it could help to reflect the disease’s nature, behavioral disturbances, and give new insight into the treatment target.

### Limitations

This study provided preliminary brain functional findings for adolescent MDD with suicidal attempt and had some limitations. First, the sample size of this study was small and future studies with larger sample sizes are needed to validate the reproducibility and generalizability of the findings. Second, this study was a cross-sectional design, which could not assign causality. Hence, longitudinal studies are critical for identifying the causal direction of the relationship to deeply elucidate the neurobiological mechanisms. Third, the proportion of female patients exceeded that of male patients. Although this may have reflected clinical epidemiology, it could limit the generalizability of findings to males. Fourth, confounding effects of puberty stage, intelligence quotient, socioeconomic status and comorbid anxiety could also have limited the interpretation and translational application of the findings. Fifth, this study aimed to delineate functional brain network alterations associated with suicidal attempts in adolescents with depression. However, the absence of a comparison group of depressed adolescents without a suicidal attempt precludes definitive interpretation; the observed abnormalities cannot be attributed specifically to suicidality. Sixth, a modest correlation was observed between SMA-inferior parietal gyrus (IPL) connectivity and HAMD scores; however, it did not survive FDR correction, necessitating further research to explore how altered brain connectivity relates to clinical severity.

## 5. Conclusions

Overall, our study revealed brain functional abnormalities in the DMN, CCN, SMN and VN of adolescent MDD with suicidal attempt, which was moderately associated with symptom severity; this suggested a possible neural basis for emotional, cognitive, and behavioral disruptions in patients, and provided imaging evidence for psychoneurological mechanisms underlying suicidal attempt in depression and potential treatment targets for future research.

## Availability of Data and Materials

All the code and data supporting the results of this study can be obtained from the corresponding authors upon reasonable request.

## Supplementary Material

Supplementary material associated with this article can be found, in the online version, at https://doi.org/10.31083/AP48336.
