# Supplementary material for: Brain Functional Network Alterations in Adolescents With Major Depressive Disorder and Suicidal Attempt: A Resting-State fMRI Study
Source: Alpha Psychiatry. 2026 Apr 22;27(2):48336. doi: 10.31083/AP48336 (PMC13156051; doi:10.31083/AP48336)
Supplement: Supplementary file 1 [file 2757-8038-27-2-48336-s1.zip › Supplementary Material.docx]

**Supplementary Materials**

*Functional connectivity results of subgroup analyses*

The results for female participants were completely consistent with the results of whole-group analysis (Supplementary Table 1). Results for the male subgroup were largely consistent with the results of whole-group analysis, except that reduced FC was not observed in several regions among male patients (Supplementary Fig. 1).

Supplementary Table 1. Differences of functional connectivity between female patients and healthy controls.

| Anatomical Regions | | FC (Mean±SD) | | p value |
| --- | --- | --- | --- | --- |
|  |  | Suicidal MDD | HC |  |
| Precentral_R | Frontal_Sup_Orb_R | 0.05±0.17 | 0.27±0.18 | ＜0.001 |
| Precentral_R | Precuneus_R | 0.12±0.24 | 0.39±0.28 | ＜0.001 |
| Supp_Motor_Area_R | Lingual_R | 0.08±0.21 | 0.23±0.16 | 0.005 |
| Precentral_R | Cuneus_L | 0.13±0.19 | 0.32±0.25 | 0.001 |
| Supp_Motor_Area_R | Supp_Motor_Area_L | 0.02±0.18 | 0.25±0.24 | ＜0.001 |
| Rolandic_Oper_L | Lingual_R | 0.02±0.17 | 0.17±0.14 | 0.001 |
| Cingulum_Mid_L | Frontal_Mid_L | 0.28±0.26 | 0.52±0.20 | ＜0.001 |
| Supp_Motor_Area_L | Parietal_Inf_R | -0.13±0.18 | 0.08±0.16 | ＜0.001 |
| Supp_Motor_Area_L | Parietal_Inf_R | -0.07±0.19 | 0.17±0.18 | ＜0.001 |
| Supp_Motor_Area_L | Parietal_Inf_L | -0.02±0.19 | 0.17±0.18 | ＜0.001 |
| Cuneus_R | Putamen_L | 0.15±0.18 | 0.31±0.16 | 0.001 |
| Frontal_Mid_R | Cerebelum_L | 0.14±0.11 | 0.27±0.20 | 0.003 |
| Cingulum_Mid_R | Precentral_L | 0.16±0.22 | 0.38±0.21 | ＜0.001 |
| Frontal_Mid_R | Cerebelum_L | 0.06±0.14 | 0.21±0.14 | ＜0.001 |

Abbreviations: Cerebelum, cerebellum; Cingulum_Mid, middle cingulate cortex; FC, functional connectivity; Frontal_Mid, middle frontal gyrus; Frontal_Sup_Orb, orbital superior frontal gyrus; HC, healthy control; L, left; Lingual, lingual gyrus; MDD, major depressive disorder; Parietal_Inf, inferior parietal lobule; Precentral, precentral gyrus; R, right; Rolandic_Oper, rolandic operculum; Supp_Motor_Area, supplementary motor area; SD, standard deviation.

Supplementary Fig. 1. Individual data distributions of functional connectivity in male participants.


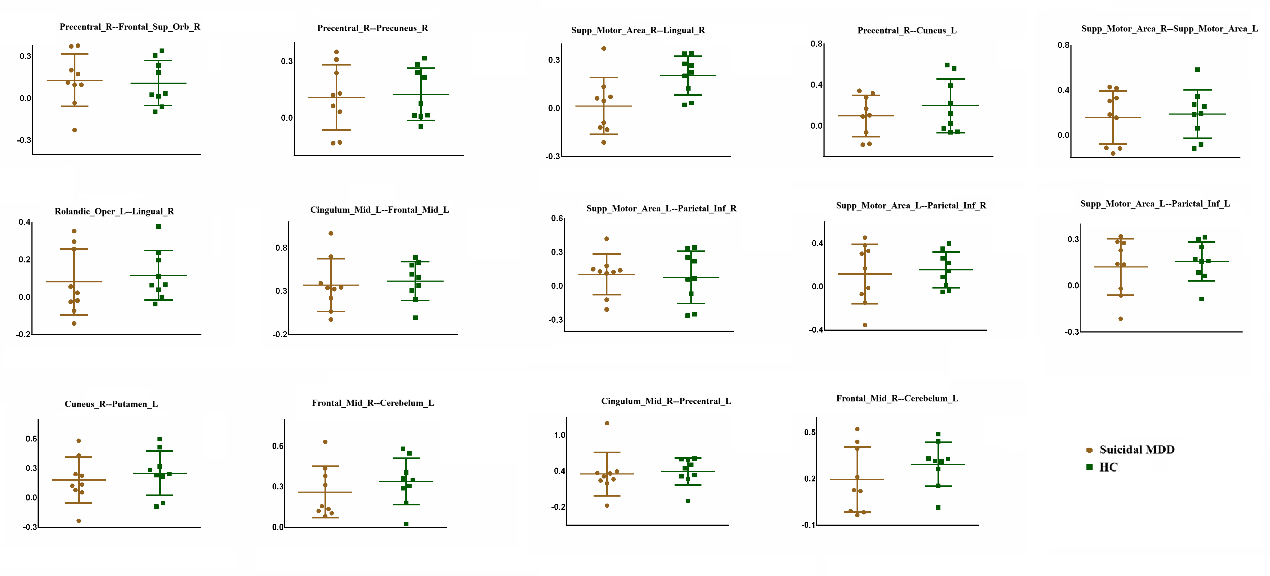
Functional connectivity was reduced or showed the tendency of decrease in male patients in the SMN, DMN, CCN and VN, including the right supplementary motor cortex-right lingual gyrus, the right precentral gyrus-left cuneus, the left rolandic operculum-right lingual gyrus, the left middle frontal gyrus-left middle cingulum cortex, the left supplementary motor cortex-bilateral inferior parietal lobule, the right cuneus-left putamen, the right middle frontal gyrus-left cerebellum.

Abbreviations: Cerebelum, cerebellum; Cingulum_Mid, middle cingulate cortex; Frontal_Mid, middle frontal gyrus; Frontal_Sup_Orb, orbital superior frontal gyrus; HC, healthy control; L, left; Lingual, lingual gyrus; MDD, major depressive disorder; Parietal_Inf, inferior parietal lobule; Precentral, precentral gyrus; R, right; Rolandic_Oper, rolandic operculum; Supp_Motor_Area, supplementary motor area.

*Graph theory analysis results of subgroup analyses*

The results of subgroup analyses for both females and males were consistent with the overall group result, showing decreased Dc in the right precentral gyrus in patients (Supplementary Fig. 2).


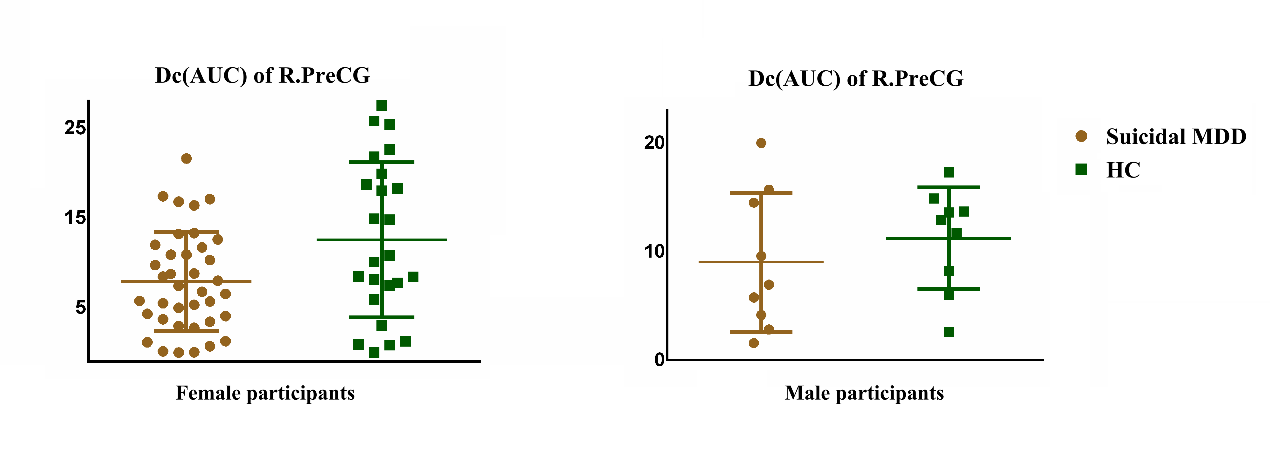
 Supplementary Fig. 2. Decreased degree centrality in the right precentral gyrus of both female and male patients.

Abbreviations: AUC, area under the curve; Dc, degree centrality; HC, healthy control; MDD, major depressive disorder; PreCG, precentral; R, right.
